# Interactions of Serum Amyloid A Proteins with the Blood-Brain Barrier: Implications for Central Nervous System Disease

**DOI:** 10.3390/ijms25126607

**Published:** 2024-06-15

**Authors:** Michelle A. Erickson, Anvitha P. Mahankali

**Affiliations:** 1 Geriatric Research Education and Clinical Center, VA Puget Sound Healthcare System, Seattle, WA 98108, USA; 2 Department of Medicine, Division of Gerontology and Geriatric Medicine, University of Washington School of Medicine, Seattle, WA 98104, USA; apm27@uw.edu

**Keywords:** serum amyloid A, blood–brain barrier, neuroinflammation, Alzheimer’s disease, lipoproteins, amyloidosis

## Abstract

Serum amyloid A (SAA) proteins are highly conserved lipoproteins that are notoriously involved in the acute phase response and systemic amyloidosis, but their biological functions are incompletely understood. Recent work has shown that SAA proteins can enter the brain by crossing the intact blood–brain barrier (BBB), and that they can impair BBB functions. Once in the central nervous system (CNS), SAA proteins can have both protective and harmful effects, which have important implications for CNS disease. In this review of the thematic series on SAA, we discuss the existing literature that relates SAA to neuroinflammation and CNS disease, and the possible roles of the BBB in these relations.

## 1. Introduction

Serum amyloid A (SAA) proteins are a family of apolipoproteins that are best known for their upregulation by the liver in response to inflammatory stimuli, and deposition in major organs during systemic amyloidosis—a rare complication of inflammatory disease [1]. SAA was first isolated from spleens and livers of subjects with secondary amyloidosis in the 1960s and subsequently characterized as a 12 kDa protein whose amino terminus was identical to that of the AA protein that comprised systemic amyloid deposits [2,3,4]. In humans, there are three SAA genes that encode protein products: *SAA1*, *2*, and *4* that are located in a 150 kilobase cluster on chromosome 11. *SAA1* and *SAA2* genes are located within 15–20 kilobases of each other and share over 90% identity and similar regulatory regions in their promoters such as NF-κB and NF-IL-6 binding sites that are activated by cytokine signaling. Both genes encode 104 amino acid proteins, and different isoforms in humans have been reported for *SAA1* and *SAA2* [5]. Because of their similarity and close genomic proximity, *SAA1* and *2* are thought to have arisen from a gene duplication. *SAA4* is located nine kilobases downstream of *SAA2*, but lacks most of the promoter regulatory regions involved in inflammatory signaling. *SAA4* encodes a longer, 112 amino acid protein which only shares about 52–55% identity with SAA1 and SAA2 and is constitutively expressed. Except in rare cases of mutations, SAA4 does not form AA amyloid [6]. SAA4 also has N-glycosylation sites towards its C-terminal end, and circulates as glycosylated and non-glycosylated forms [7]. In humans, *SAA3* is thought to be a pseudogene and its protein expression has generally not been detected [8]. In mice, *Saa* genes are organized on chromosome 7 similarly to human *SAA* genes [9], except that *Saa3* is expressed as a functional protein in mice. Saa3 is often referred to as “extrahepatic” because its upregulation can be detected in many tissues in addition to liver in response to inflammatory insults [1,10,11]. Although predominantly induced by the liver with inflammation, Saa1 and Saa2 can also be expressed extrahepatically [12,13]. SAA/Saa1-3 are considered to be acute phase proteins because they are upregulated by inflammatory stimuli. SAA/Saa4 is constitutively expressed in humans and mice and is upregulated to a lesser extent with inflammation vs. acute phase SAAs. Under basal conditions, SAA4 is likely the major SAA isoform in the circulation, but its functions are not well understood [10,14]. All SAA proteins are incorporated into high-density lipoproteins (HDL) [10,14]. Similar to C-reactive protein (CRP), acute-phase SAA proteins (i.e., SAA1 and SAA2) are sensitive biomarkers for a range of diseases where inflammation is implicated [15]. The biological functions of SAA have been extensively studied, but more recent studies in SAA knockout or overexpressing mice have indicated that recombinant and endogenously produced SAA may function differently [16]. Given its amyloidogenic properties and inflammatory involvement, SAA has also been studied in the context of Alzheimer’s disease, multiple sclerosis, stroke, traumatic brain injuries, mood disorders, and other CNS conditions. As SAA is largely produced systemically [17], recent work showing that Saa1 and 2 can cross the intact blood–brain barrier (BBB) in vivo [18] offers insight into neuro-immune communication mechanisms of SAA. In this review, we discuss the systemic and CNS functions of SAA, the involvement of the BBB, and the participation of SAA proteins in CNS diseases. 

## 2. Systemic Functions of SAA Proteins

The acute phase response is a core part of the innate immune system and involves the modulation of protein secretion by hepatocytes in the liver. This results in changes in circulating protein profiles: positive acute phase proteins are those whose expression increases, and negative acute phase proteins are those whose expression decreases during an acute phase response [19]. The acute phase response occurs widely across animal species, but the profiles and quantities of proteins secreted differ across species [20]. For example, in humans, SAA and CRP are the major acute phase proteins, whereas in mice, these are SAA, serum amyloid P, and haptoglobin. In rats, isoforms of SAA are not potently upregulated as a component of the acute phase response [20]. Therefore, the majority of SAA functions have been studied in mice and humans. SAA (particularly, SAA1 and 2) is among the most abundant of acute phase proteins, as it has been estimated that about 2.5% of the protein expression in the liver is diverted to SAA synthesis during the acute phase response [21]. Isoforms of acute phase SAA proteins are largely regulated by pro-inflammatory cytokines such as interleukins (IL)-1 and 6, as well as tumor necrosis alpha (TNF-α) in the liver [20,22]. Other cytokines in addition to IL-6 that signal, in part, through GP130 including IL-11, ciliary neurotrophic factor (CNTF), leukemia inhibitory factor (LIF), oncostatin M (OSM), and cardiotrphin-1 (CT-1) have been shown to induce SAA when given intravenously [23]. Although CNTF potently inhibits bacterial lipopolysaccharide (LPS)-induced TNF-α in the brain and periphery and prevents LPS-induced mortality in mice, LPS-induced SAA levels are not altered by CNTF [24]. It has been shown for IL-1β and CNTF that injection in the cerebral ventricle induces a stronger liver acute phase response/SAA upregulation vs. injection via peripheral routes, indicating that neuroinflammation is a particularly potent inducer of systemic SAA [25,26]. Glucocorticoids also contribute to SAA upregulation, and the nature of SAA expression control can vary by cell type [27], although the liver is the predominant source of SAA in the blood. 

The functions of SAA in the body are somewhat controversial in the literature, because many of the functions ascribed to SAA have been from studies using recombinant forms [28]. More recent studies comparing recombinant vs. endogenous SAA have revealed different functions [16,28,29,30], and in some cases it was found that recombinant SAA produced in bacteria contained impurities that were the main cause of inflammatory effects [31,32,33]. It is also apparent that the functions of SAA differ depending on whether SAA is associated with high-density lipoproteins (HDL) [34]. In blood, it is estimated that about 95% of SAA from the liver is bound to HDL [14]. Thus, the functions of SAA that have been shown in vivo using knockout or overexpression mouse models are mainly considered in this section. Some of these include the facilitation of bacterial clearance, regulation of cellular immune responses and of HDL functions (discussed in the next paragraph), and contribution to the progression of atherosclerosis, which are summarized in detail elsewhere [28]. SAA expression may also be regulated in part by the microbiome, as SAA along with pro-inflammatory cytokine expression are reduced in germ-free mice following LPS treatments, rendering them less susceptible to LPS [35]. SAA may also have metabolic functions, as treatment with anti-SAA antisense in vivo prevented weight gain and insulin resistance, as well as metabolic endotoxemia in mice on a high-fat diet [36]. However, mice lacking expression of *Saa1-3* show a similar phenotype as wild-type mice when fed an obesogenic diet, including similar levels of weight gain and insulin sensitivity, although the *Saa1-3* knockout mice on obesogenic diet had significantly impaired glucose tolerance vs. wild-type counterparts [37]. Mice expressing human SAA1 in adipose tissue also showed no evidence of insulin resistance or obesity-related inflammation [38]. SAA is thought to signal through a number of pattern recognition receptors, although some SAA receptors such as toll-like receptors (TLRs) 2 and 4 have been contested as true SAA receptors due to bacterial impurities in recombinant preparations that also activate these receptors [28]. Signaling through scavenger receptors such as the formyl peptide receptor-2 (FPR-2), scavenger receptor B2 and B3, CD36, and the receptor for advanced glycation end-products are also supported [28].

As stated above, a large proportion of SAA is incorporated into HDL after it is synthesized; lipidation of SAA in hepatocytes is medicated via the cholesterol efflux regulatory protein ABCA1 [39]. When incorporated into HDL, the pro-inflammatory functions of SAA in vivo appear to be mitigated. This is supported in an inducible SAA overexpression model, where induced SAA expression is predominantly incorporated into HDL and induced at levels reflective of a major acute phase response. In this model, circulating serum amyloid P is not upregulated, indicating that SAA overexpression does not induce an acute phase response in mice. However, systemic amyloidosis does occur in this model, indicating that AA amyloidosis can occur with SAA overexpression in the absence of inflammation [40]. Similarly, it was shown in vivo that HDL injections mitigated the vascular and renal dysfunctions caused by repeated injections of recombinant SAA [41], and that lipidated or in vivo overexpressed SAA does not alter cytokine levels such as G-CSF [16]. HDL also inhibits SAA-mediated production of reactive oxygen species and NLRP3 inflammasome activation [42]. However, SAA can dissociate from HDL via different mechanisms, resulting in a small proportion of lipid-poor circulating amyloid which may have more pronounced pro-inflammatory functions that are similar to those described in studies using recombinant SAA. Poorly lipidated SAA may also arise when it is expressed in tissues outside of the liver [14]. The regulatory effects of SAA on HDL functions have also been studied. One important function of HDL is reverse cholesterol transport, which is a mechanism by which HDL removes cholesterol from peripheral tissues and delivers it to the liver, where it is either removed by the gallbladder or redistributed to tissues [43]. Effects of SAA on reverse cholesterol transport are controversial, with some studies showing that it enhances and others showing that it inhibits this function of HDL [14]. Studies have also shown that SAA can both impair and promote antioxidant and anti-inflammatory functions of HDL, depending on the experimental context [14]. SAA can be exchanged from HDL to other lipoproteins and can regulate interactions of HDL and other lipoproteins with proteoglycans, particularly those of the vasculature [44,45]. Studies have also indicated that Saa1 vs. Saa4 have some differences in their ability to alter lipoproteins, with Saa4 overexpression increasing LDL and triglycerides [46]. Thus, SAA functions appear to be largely regulated based on associations with lipoproteins.

## 3. Brain-Related Functions of SAA Proteins

In this section, we focus on the known functions of SAA in the brain, as well as its associations with and potential contributions to neuroimmune communication. To explore the literature on brain-related functions of SAA proteins, a PubMed search of ““serum amyloid A” and brain” on 30 March 2024 produced 234 results with publications ranging from 1980 to 2024. We further screened these titles and abstracts and excluded articles that were not original research, were not published in English, or otherwise did not inform any relation of SAA to CNS functions. After these exclusions, the number of relevant articles was 136. These articles were then sorted into those of biomarker studies (39 articles) which predominantly evaluated SAA as a biomarker for stroke/white matter disease and traumatic brain injury, articles on the tissue locations of expression and deposition of SAA proteins and AA amyloid produced by them (13 articles), studies on the regulation of SAA expression (27 articles), SAA molecular interactions and signaling (4 articles), SAA physiologic functions (22 articles), and the involvement of SAA in CNS diseases (31 articles), particularly Alzheimer’s disease. These articles identified from this search highlight the general scope and contexts in which brain-related functions of SAA have been studied. We discuss many of these articles from our search in depth below, particularly focusing on those which evaluate functions of endogenous or genetically/pharmacologically modified SAA in healthy and disease states.

Many of the early works relating SAA proteins to the brain involved investigations of AA amyloid deposition in systemic AA amyloidosis, which is a rare complication of some inflammatory diseases. In general, AA amyloid is not found to deposit in the brain or brain vasculature, although amyloid beta immunoreactivity has been detected and CNS complications associated with AA amyloidosis have been reported [47,48]. However, the neuronal overexpression of mouse Saa1 can, specifically under inflammatory conditions, result in SAA deposition as amyloid in the brain parenchyma and vasculature, which is exacerbated with age [49]. A study of *Saa1* promoter activity identified that its extrahepatic expression is upregulated by about 0.5–2-fold in the mouse brain following injection with LPS or TNF-α, although upregulation in the brain was generally many orders of magnitude lower than most other organs, with the exception of the spleen [17]. The reason for lower levels of SAA promoter activity in brain vs. other tissues is not known but could be related to many factors including local pro-inflammatory cytokine concentrations, differences in activation of downstream signaling pathways, epigenetic regulation of gene expression, or post-transcriptional mechanisms. Increases in SAA expression following inflammatory or traumatic insults are also supported to differ by sex. In the same study of Saa1 promoter activity, males were found to have higher promoter activity vs. females in the liver, lungs, intestines, and heart after intraperitoneal injection of either LPS or TNF-α. Along similar lines, males generally had higher SAA levels in plasma vs. females in the early stages (6 h) following traumatic brain injury, and higher SAA protein levels in brain 1 day post-injury [50]. Increased mRNA detection of *Saa1* in brain was localized in astrocytes, microglia, and in blood vessels and reached maximal levels 1–3 days post-traumatic brain injury [50].

SAA is increased in plasma following stroke, and in a mouse model of cerebral ischemia/reperfusion injury, SAA was maximally elevated between 24 and 60 h post-stroke injury and persisted to 120 h. *Saa1* and *2* double knockout significantly reduced IL-1β, TNF-α, and transforming growth factor (TGF)-β proteins in the brain, cerebral infarct volume and tunnel staining, and reactive gliosis, and improved survival and cognitive functions. Interestingly, *Saa3* knockout did not have a significant effect on these parameters [51]. The peroxisome proliferator-activated receptor (PPAR)-α activator, fenofibrate, which has poor BBB penetration reduced Saa1 synthesis by the liver and also neutrophil infiltration in both the liver and the brain [52]. It was also found that an SAA-blocking antibody mitigated the brain damage and neurological impairments in mice with brain ischemic-reperfusion injury [51]. These findings suggest that peripheral mitigation of SAA could be protective in brain injury, although this is a cautious interpretation since BBB leakage could also contribute to brain entry of these therapeutics where they may have local protective effects.

Exposure to air pollutants, such as ozone, have been identified as a risk factor for cognitive decline and Alzheimer’s disease (AD) [53,54,55] and to mechanistically contribute to AD-associated CNS and peripheral responses in AD mouse models [56]. *Saa1* and *2* knockout has been shown to mitigate the weight loss in a mouse model of ozone-induced lung inflammation, although SAA knockout mice did not have reductions in lung inflammation, systemic increases in kynurenine, or depressive-like behavior [57]. Of note, systemic and CNS elevations of SAA following ozone exposure occurred in the absence of pro-inflammatory cytokine upregulation in blood or brain, and so the knockout data suggest a cytokine-independent role of SAA in mediating weight loss [18]. Independently of air pollutant exposure, chronic overexpression of SAA1 by the liver contributes to depressive-like behaviors in mice [58]. Thus, acute and chronic increases in SAA contribute, in part, to inflammation-associated weight loss and depressive-like behaviors, respectively.

In contrast, Saa3 in the brain may have some protective effects, as it has been shown to prevent the hyperphosphorylation of the Alzheimer’s protein tau in response to LPS, possibly through the induction of the anti-inflammatory cytokine IL-10 in microglia [59]. Saa3 was further shown to be upregulated in brains of APP/PS1 transgenic mice, which deposit amyloid beta plaques in their brains and are thus considered a model of AD. In APP/PS1 mice lacking Saa3, astrocyte activation around amyloid plaques was worse, which was supported by in vitro studies to be attributed to the inhibitory effects of Saa3 on astrocyte migration through activation of p38 MAPK signaling [60].

**Figure 1:**
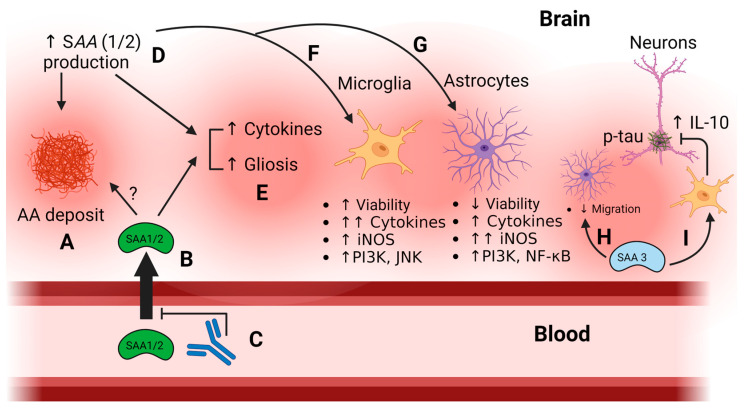
Inflammation-related functions of SAA proteins in the brain. Up arrows indicate an increase and down arrows indicate a decrease, the number of arrows indicates the relative intensity of the change. (A) SAA may deposit in brain as AA aggregates under inflammatory conditions when SAA levels are high [49]; (B) SAA1/2 is transported across the intact BBB [18,58]; it is unclear whether SAA from blood contributes to AA aggregates in the brain, indicated by the “?”. (C) Anti-SAA antibodies inhibit SAA accumulation in the brain [51]; (D) inflammatory stimuli can increase local expression of SAA in the brain [17]; (E) brain elevations of SAA1/2 contribute to increased brain cytokines and gliosis [51,61] and differently affect (F) microglia and (G) astrocytes [61]. Functions of Saa3 in the brain appear to differ from those of SAA1/2, with Saa3 being shown to (H) suppress astrocyte migration [60] and (I) stimulate anti-inflammatory IL-10 production in microglia, preventing tau phosphorylation in neurons [59]. Figure created with BioRender.

Treatments with recombinant SAA in vitro modulate the functions of astrocytes and microglia cells, decreasing the viability of astrocytes and increasing viability of microglia. Cytokines and inducible nitric oxide synthase (iNOS) were also differentially induced in both cell types. Phosphoinositide 3-kinase (PI3K) was a common pathway of SAA-mediated effects in astrocytes and microglia, whereas the c-Jun N-terminal kinase (JNK) pathway was selectively induced in microglia and the nuclear factor kappa-light-chain-enhancer of activated B cells (NF-kB) pathway selectively activated in astrocytes [61]. The functions of SAA proteins in the brain described above are illustrated in Figure 1. Similar to systemic SAA regulation, it is possible that HDL may also regulate SAA activities in the brain, although this has not been formally tested. Brain cholesterol and its lipoproteins are almost exclusively produced via local synthesis, mainly by astrocytes and oligodendrocytes [62]. However, certain oxysterols such as 24S-hydroxycholesterol and 27-hydroxycholesterol can cross the BBB [63]. HDL is the most abundant lipoprotein in the brain and is mainly produced by astrocytes [63]. However, there is some evidence for lipoprotein exchange across brain barriers, particularly ApoAI across the choroid plexus [64] which may contribute to its inclusion in cerebrospinal fluid (CSF) lipoprotein particles [52]. The transport of SAA across the blood–brain barrier is discussed in the next section. Overall, the involvement of HDL in the regulation of CNS SAA functions remains an understudied area.

## 4. Evidence of SAA Interactions with the Blood–Brain Barrier (BBB)

The BBB is a specialized property of brain endothelial cells which both protects against the unregulated leakage of substances from blood into brain, and regulates the transport of vital substances to the brain that cannot passively permeate such as glucose, transferrin, and insulin [65]. The barrier properties of the BBB are conferred in part by endothelial expression of highly impermeable tight junction proteins, which limit the leakage of substances between cells. Specialized lipid transporters, such as Mfsd2a, that are enriched in brain endothelial cells prevent bulk-phase transcytosis by altering the membrane lipid composition, which suppresses caveolae formation [66]. Transport of substances across the BBB is regulated by a variety of transporters. Examples include carrier proteins that are energy-independent (e.g., the glucose transporter GLUT-1), transporters that undergo receptor-mediated transcytosis (e.g., transferrin receptor) after the binding of their substrates [65], and efflux pumps like P-glycoprotein [67]. The BBB is also an important interface for neuroimmune communication, regulating the “immune privileged” status of the CNS. Many examples of immune-active substances crossing the intact BBB have been described, including transport of pro-inflammatory cytokines, chemokines, and viral proteins [68,69,70,71,72]. Thus, it is not surprising that SAA is among proteins that can cross the intact BBB. Our prior work has shown that both mouse Saa1 and Saa2 injected intravenously can cross the intact mouse BBB, and that CNS SAA is likely derived mainly from the periphery in a mouse model of ozone exposure since its RNA expression is not increased in the brain [18]. Similarly, another group showed that mouse Saa1 that was specifically overexpressed in the liver was shown to cross the intact BBB to enter the brain and contribute to depressive-like behaviors in mice [58]. When the Saa1-liver overexpressing mouse was crossed with an APP transgenic mouse model of AD, it was found that more Aβ and Saa1 accumulated in the brain in the double transgenic, and that there was more IL-6 induced in brain and serum. The double transgenic mice also performed worse on object recognition memory [73]. However, when SAA was experimentally upregulated by LPS in either wild-type or Tg2576 mice which develop amyloid plaques, no differences were found in CNS SAA levels in older Tg2576 mice vs. controls 24 h later [74], suggesting that CNS Aβ deposits do not acutely alter the accumulation of endogenously expressed SAA in the brain. In addition to transport in the blood-to-brain direction, substances can cross the intact BBB in the brain-to-blood direction, which is an important mechanism for the clearance of substances such as amyloid beta from the brain [75]. Presently, it has not yet been determined whether SAA has a BBB efflux system, although it has been shown that the overexpression of SAA in the CNS does not change the concentrations of circulating SAA [49], suggesting that SAA expressed in the brain is largely retained or degraded locally, rather than being effluxed across the BBB where it would be expected to be detectable in blood, if intact. The transport of Saa3 or Saa4 across the BBB has not been evaluated to date.

**Figure 2:**
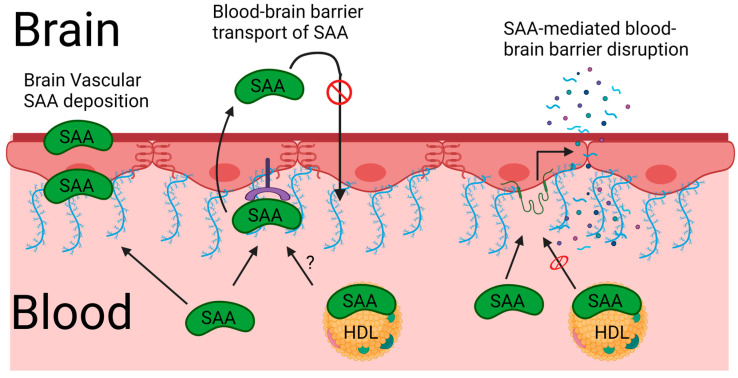
Interactions of serum amyloid A (SAA) proteins with the vascular blood–brain barrier. The processes of vascular SAA deposition, SAA transport across the intact BBB, and SAA-mediated BBB disruption are depicted. The pink cell layer depicts brain endothelial cells with the dark red line on the brain-facing side depicting the basement membrane. The blue projections from endothelial cells represent the glycocalyx. The purple projection from an endothelial cell depicts a transporter of SAA whose identity is not yet determined. The red structures between endothelial cells represent tight-junction proteins. The green projection from the endothelial cell represents a receptor through which SAA mediates BBB disruption. It is unclear whether SAA that circulates bound to HDL can cross the intact BBB, as indicated by the question mark (?) above the arrow depicting this process. It has been shown that HDL inhibits SAA-mediated disruption of the BBB [76], as indicated by the red circle-slash over the arrow depicting this process. SAA overexpression in the brain does not result in increased SAA levels in the circulation [49], suggesting that brain SAA is not transported in the brain-to-blood direction, indicated by the red circle-slash over the arrow depicting this direction of transport. Figure created with BioRender.

There is also evidence that SAA interactions with the BBB can result in its leakage. This has been shown in vitro using recombinant Apo-SAA treatments of primary rat brain endothelial cells in a transwell culture system [76]. SAA significantly reduced the transendothelial electrical resistance and leakage to the small fluorescent tracer, sodium fluorescein, which was associated with significantly reduced expression of the tight junction protein marker Claudin-5, but not ZO-1 or Occludin [76]. These data thus suggest that interactions of recombinant SAA with brain endothelial cells could lead to the loss of tight junction proteins and increased leakage between cells. HDL was shown to inhibit these effects of Apo-SAA on the in vitro BBB [76]. Thus, the ability of SAA to induce BBB disruption in vivo may depend on free circulating concentrations. It is likely that endogenously expressed SAA is limited in its ability to disrupt the BBB since most of it circulates as a component of HDL. There are also examples where strong systemic upregulation of SAA, such as that which occurs with lower doses of bacterial LPS can occur in the absence of overt BBB disruption [77]. In other words, total SAA elevations in vivo are not synonymous with BBB leakage. However, routine SAA assays generally do not distinguish that which is HDL-bound vs. unbound in blood, and so the free pool of SAA available to interact with the BBB has not been defined in most studies. Another consideration is that the inflammatory response to pathogens is generally adaptive and entails a highly evolved process that enables the otherwise healthy host to return to their homeostatic baseline once the pathogen is cleared. Inflammatory responses to traumatic insults are often associated with lasting damage, and evidence supports that SAA upregulation occurs in a variety of CNS injuries, such as traumatic brain injury or cerebrovascular accidents [51,78,79]. In mice, it has been shown that Saa1 and 2 contribute to BBB leakage in a stroke model, as Evans blue extravasation into the brain at 6 and 12 h following ischemia-reperfusion was reduced in Saa 1, Saa2, and double Saa 1 and 2 knockouts, but not in an Saa3 knockout [51]. Thus, the extent of BBB disruption appears to depend on the pathophysiologic context but the molecular conditions that determine when SAA does or does not cause BBB dysfunction have not yet been clearly demonstrated in vivo. Whether Saa4 contributes to BBB leakage has not been investigated. The finding that SAA can contribute to BBB leakage could be relevant to a number of conditions where SAA is found to deposit in the brain vasculature. For example, SAA was found to deposit in cerebral capillaries and microinfarcts of hypertensive monkeys [80]. SAA deposition was also reported in human saccular intracranial aneurisms and the amount of SAA deposition was positively associated with wall degeneration and rupture and proximal inflammation [81]. The extent to which cerebrovascular deposition of SAA occurs in other CNS conditions in which it is implicated (e.g., traumatic brain injury, Alzheimer’s disease, etc.) has not yet been reported and remains an open area of study. Figure 2 summarizes these interactions of SAA proteins with the vascular BBB.

Possible interactions of SAA proteins with the BBB may also be inferred from studies of the peripheral vasculature. As noted earlier, acute phase SAA can be expressed and upregulated extrahepatically, and human coronary artery endothelial cells are among cells that produce SAA1,2, and 4 and can upregulate SAA1 and 2 expressions in response to cytokines [82]. Recombinant SAA can stimulate the migration and proliferation of endothelial cells [83]. The neutralization of Saa1 and 2 in vivo has been shown to protect sinusoidal endothelial cells from damage and platelet aggregation due to acetaminophen toxicity [84]. Similarly, SAA4 has been found to be upregulated in peripheral atherosclerotic lesions, and recombinant SAA4 has procoagulant activity [85,86]. Whether such interactions with the brain vasculature occur remains to be determined.

## 5. Evidence for the Involvement of SAA Proteins in CNS Diseases

SAA has been studied in the context of AD, stroke, traumatic brain injury, multiple sclerosis, brain cancers, and depression. In many of these conditions, SAA elevations have been detected in serum, plasma, or CSF, thus highlighting its utility as a possible biomarker of disease severity or prognosis [78,87]. A notable example is that of the balloon occlusion test, which is a clinical test of brain collateral blood flow. In human subjects receiving this test, SAA was found by proteomic methods to be upregulated, indicating that the blockage of cerebral blood flow, even when transient, is sufficient to induce systemic rises in SAA [79]. As for its mechanistic functions in CNS disease, we have discussed some examples of studies in mouse models, such as SAA involvement in stroke outcomes, and in AD models in Section 2 and Section 3. SAA has been studied most extensively in AD, particularly when it was realized that CNS amyloid deposition was an AD hallmark. Early studies found no immunoreactivity of antisera against systemic AA amyloid in AD brains and determined that AA amyloids and cerebral amyloids (now known to be predominantly comprised of amyloid beta protein) were distinct entities [88,89]. However, later studies showed that SAA was present on HDL in CSF of AD and non-AD subjects and was significantly elevated in AD CSF and plaques [90]. SAA was found to be localized in AD to the myelin sheaths of axons, which was attributed to its cholesterol-binding properties [91]. Increased local production of SAA in the CNS was also noted in AD [92], as were slight elevations in plasma [93]. Interrelated mechanisms that have been suggested to link SAA and AD include lipid dyshomeostasis and altered immune cell functions. 27-hydroxycholesterol (27-OHC) is a cholesterol metabolite that was found to be increased in brains of subjects with mild cognitive impairment (MCI), a prodromal condition of AD. Mice treated with 27-OHC had increased SAA in their brains, which was associated with altered Th17/Treg balance that was dependent on RORγt [94]. It has also been shown that SAA1 overexpression in an AD mouse model that forms amyloid beta plaques results in worse plaque deposition, gliosis, and memory impairment [73]. However, it should be noted that accumulation of amyloids in the brain can have protective effects. For example, the accumulation of amyloid beta in AD has been associated with anti-microbial activities and so may be a mechanism by which the brain protects itself from pathogens [95]. Whether SAA could confer protection via amyloidogenic mechanisms in AD is presently unclear.

SAA has also been implicated in the pathogenesis of multiple sclerosis (MS). The acute phase response in experimental autoimmune encephalomyelitis (EAE), a rodent model of MS, was shown to precede onset of clinical disease symptoms and included upregulation of Saa1 and 2 by the liver. EAE mice in the remission phase also have more pronounced upregulation of SAA in the liver and brain in response to LPS, compared to control mice treated with only adjuvant [96]. It has also been shown in an EAE model that a Saa3+ monocyte subset derived from early myeloid progenitors was contributing to MS pathogenesis [97]. To date, SAA knockout, inhibition, or overexpression has not been evaluated in MS, but studies such as these could further identify their participation in MS pathogenesis. Overall, the involvement of SAA proteins in CNS diseases is likely to be important but remains understudied.

## 6. Conclusions

SAA proteins have complex and nuanced functions in the CNS and periphery and are likely participants in CNS disease pathogenesis. Interactions of SAA proteins with the BBB, including their ability to be transported from blood to brain across the intact BBB and to cause BBB leakage are likely integral to their functions in the CNS. The functions of SAA depend, in part, on their lipidation and incorporation into HDL and other lipoprotein particles. HDL appears to prevent SAA-mediated BBB disruption, but its regulation of SAA functions in the brain has not been studied and so reflects an important knowledge gap in the field. The isoform-specific effects of SAA proteins in the brain are also nuanced. The current literature supports that Saa1 and 2 are mainly pathogenic, whereas Saa3 has both protective and harmful effects, and Saa4 is essentially unstudied, highlighting another potentially important unexplored area of SAA research. Therefore, mechanistic involvement of SAA proteins in neuroimmune communication, CNS diseases, and CNS disease risk factors, such as metabolic syndrome, gut dyshomeostasis, and air pollution exposure, requires further study, particularly in transgenic rodent models where endogenous and tissue-specific functions of SAA proteins can be evaluated. The endogenous and disease-related functions of SAA proteins remain nuanced, and so additional comprehensive studies that characterize their overall and isoform-specific functions, HDL-dependent and -independent effects, predominant tissue sources, and receptor-mediated signaling in vivo are needed. As Saa3 is expressed in mice but not humans, the extent to which Saa3 has unique and overlapping functions with SAA1 and 2 is an important research question, particularly with regard to therapeutic development for human CNS diseases. Finally, the extent to which SAA is protective needs to be considered when developing therapeutic approaches aimed at lowering SAA concentrations or blocking its activities, particularly in the context of major CNS disease risk factors such as aging. In conclusion, SAA proteins have important and multifaced functions in acute systemic responses to inflammatory and traumatic insults and in CNS diseases, warranting further exploration to elucidate their precise activities and potential as therapeutic targets.

## Data Availability

No new data were created.

## Abbreviations

27-OHC	27-hydroxycholesterol
AD	Alzheimer’s disease
BBB	blood–brain barrier
CNS	central nervous system
CNTF	ciliary neurotrophic factor
CRP	c-reactive protein
CSF	cerebrospinal fluid
CT-1	cardiotrophin-1
EAE	experimental autoimmune encephalomyelitis
FPR-2	formyl peptide receptor 2
HDL	high-density lipoprotein
IL	interleukin
iNOS	inducible nitric oxide synthase
JNK	c-Jun N-terminal kinase
LIF	leukemia inhibitory factor
LPS	lipopolysaccharide
MCI	mild cognitive impairment
MS	multiple sclerosis
NF-κB	nuclear factor kappa-light-chain-enhancer of activated B cells
OSM	oncostatin M
PI3K	phosphoinositide 3-kinase
PPAR	peroxisome proliferator-activated receptor
SAA	serum amyloid A
TGF	transforming growth factor
TLR	toll-like receptor
TNF-α	tumor necrosis factor-alpha
